# Editorial Board Members’ Collection Series: QSAR and Computational Approaches to Drug Discovery

**DOI:** 10.3390/ijms27167247

**Published:** 2026-08-14

**Authors:** Francisco Torrens, Jesús Vicente de Julián-Ortiz

**Affiliations:** 1 Institut Universitari de Ciència Molecular, Universitat de València, Edifici d’Instituts de Paterna, P.O. Box 22085, 46071 València, Spain; 2 Cheminformatics Research Lab, Department of Physical Chemistry, Faculty of Pharmacy and Food Sciences, University of Valencia, Av. Vicent Andrés Estellés 22, 46100 Burjassot, Spain; jesus.julian@uv.es

Considerable effort is being devoted to better understanding and treating diseases. In this context, computational modelling and simulation are critical in biomedical research. Computer-aided drug design allows an understanding of the biomolecular interactions and the molecular mechanisms of diseases and is fundamental for the collection, processing, analysis, and modelling of data. Computer-aided drug design is also central in risk assessment, where less animal testing is necessary. Drug design involves the multi-objective optimization of efficacy, pharmacokinetics, and safety. Additionally, advances in artificial intelligence, machine learning, and deep learning have provided a basis for more effective searches of chemical spaces and predictions of bioproperties based on molecular structure. Carefully assessing the validity of results provided by these tools remains the most important matter.

We launched this Editorial Board Members’ Collection to showcase approaches that implement robust QSAR and computational design while emphasizing validation and interpretability in the core areas of interest of our groups in theoretical chemistry, molecular topology, topological indices, and virtual screening. Six contributions were published after single-anonymized peer review.

First, Soto-Delgado et al. (contribution 1) describe the 3D-QSAR integrating docking alignment and molecular dynamics for benzamide derivatives used as HDAC1 inhibitors. Ligand- and receptor-based models and electrostatic/steric contour maps show that the electron density around the benzamide ring enhances activity, with the MD energy decomposition being consistent with the QSAR.

Second, Toropova et al. (contribution 2) used optimal descriptors derived from the SMILES notation to model the acute toxicity of pharmaceuticals in fish. With five random training/validation splits (R2ext = 0.67), the work exemplifies ecotoxicological QSAR supporting the 3Rs principle for complex endpoints.

Third, a QSAR study of cathepsin L inhibitors (contribution 3) in SARS-CoV-2 entry compared a heuristic method, gene expression programming, random forest, and multi-kernel SVR (RBF, LMIX2-SVR, LMIX3-SVR) optimized with PSO. LMIX3-SVR (R2 train/test 0.9676/0.9632, R5-fold2 0.9043, Rloo2 0.9525) predicted 578 new designs, linking kernel learning to antiviral design.

Fourth, Alcolea Palafox et al. (contribution 4) report DFT (B3LYP, CAM-B3LYP, M06-2X) and experimental IR/Raman analyses of Ln(ligand)3 complexes (Ln = La, Ce, Pr, Nd, up to Er) with triazole ligands, correlating geometry, charges, and HOMO–LUMO gaps with electron/hydrogen transfer capacity (DPPH/ABTS). The findings are useful for tuning pharmacological activity.

Fifth, Pavlović Saftić et al. (contribution 5) describe trimeric and tetrameric cationic styryl dyes as fluorescence/CD probes for ds-DNA/RNA. With 350–420 nm absorption, 100–160 nm Stokes shift, and a Φf up to 0.57, small trimers show selective ICD recognition of specific nucleic acid structures and efficient live-cell uptake with negligible cytotoxicity.

Sixth, Koirala et al. (contribution 6) review AI-integrated QSAR from MLR/PLS to graph neural networks and SMILES transformers, covering docking, MD, PROTACs, ADMET, databases, and cloud platforms. They explicitly discuss validation, interpretability, regulatory and ethical challenges. This contribution has been the most viewed to date (>7900 views, 29 citations) and serves as guideline for explainable, data-rich pipelines.

Collectively, this Special Issue illustrates three requirements: (i) performing validation beyond fit using scaffold/time splits, applicability domain, and prospective testing; (ii) ensuring interpretability, coupling statistical models with MEDT, conceptual DFT, and physics-based simulations; and (iii) the use of responsible AI, with FAIR data, provenance, and risk-tiered validation for emerging foundation models. When grounded in rigorous validation and mechanistic insight, QSAR accelerates hit discovery and lead optimization without compromising safety.

We thank the authors, reviewers, and *IJMS* Editorial Office for making this Collection possible. We hope it inspires the development of reproducible models bridging mathematical chemistry, molecular topology, and experimental pharmacology.

